# Application of nanomaterials in proteomics-driven precision medicine

**DOI:** 10.7150/thno.64325

**Published:** 2022-03-06

**Authors:** Yong Zhang, Haonan Yang, Yanbao Yu, Ying Zhang

**Affiliations:** 1 Provincial Key Laboratory of Rural Energy Engineering in Yunnan, School of Energy and Environment Science, Yunnan Normal University, Kunming, China, 650500.; 2 Institutes of Biomedical Sciences and Department of Chemistry, Fudan University, China, 200032.; 3 NHC Key Laboratory of Glycoconjugates Research, Fudan University, China, 200032.; 4 Department of Chemistry and Biochemistry, University of Delaware, Newark, DE 19716, USA.

**Keywords:** Nanoparticle, nanotechnology, mass spectrometry, biomarkers, proteomics, post- translational modifications

## Abstract

Nanostructured devices and nanoparticles have fundamentally reshaped the development of precision healthcare in recent decades. Meanwhile, mass spectrometry (MS)-based proteomics has evolved from simple protein sequencing to a powerful approach that identifies disease patterns and signatures, reveals molecular mechanisms of pathological processes, and develops therapeutic or preventive drugs. Significantly, the two distinct disciplines have synergized and expanded our knowledge about human health and disease, as evidenced by a variety of nanotechnology-assisted sample processing strategies, facilitating in-depth proteome profiling and post-translational modifications (PTMs) characterization. This review summarizes recent advances in nanoparticle design for better enrichment of marker proteins and their PTMs from various bio-specimens and emerging nanotechnologies that are applied to MS-based proteomics for precision medicine discovery.

## Introduction

Precision medicine, also known as "personalized medicine", is an innovative approach to tailoring disease prevention and treatment based upon individual clinical characteristics, including genes, proteins, metabolites, environment, and lifestyle 1, 2. Multiple layers of information derived from the genome, transcriptome, and interactome are integrated to help better understand the complexity of human health and diseases that cannot be explained by any single approach 3. Proteome refers to the complete set of proteins expressed in cells, tissues, or organisms, and proteomics investigates the proteome composition and activities to obtain a system-level understanding of disease occurrence, cell metabolism, and other processes 4. Proteins are the main executors of cellular activities and are among the most critical macromolecules in precision medicine 5. Thus, developing methods to measure alterations of proteins in the context of their abundance, localization, interaction, and modifications could provide novel insights into the pathophysiology of cancer and other diseases and ultimately aid the development of precision medicine 6.

In the past decade, mass spectrometry (MS)-based proteomics has witnessed tremendous advances in accuracy, sensitivity, automation, and throughput and made remarkable contributions to novel biomarker discovery and our understanding of underlying molecular mechanisms of a variety of diseases 7, 8. More recently, proteomics has shifted its focus from the global characterization of protein expression to investigations of the spatial and temporal organization of the proteome, for instance, using targeted proteomics 9. However, challenges still exist as the proteome is dynamic and condition-dependent, leading to overwhelming complexity and heterogeneity of the abundance of proteins and their post-translational modifications (PTMs). The latter has been shown to mediate a wide variety of cellular processes, such as signal transduction and subcellular localization 10, significantly expanding the size of predicted human proteome (10-100 times) 11.

**Figure 1:**
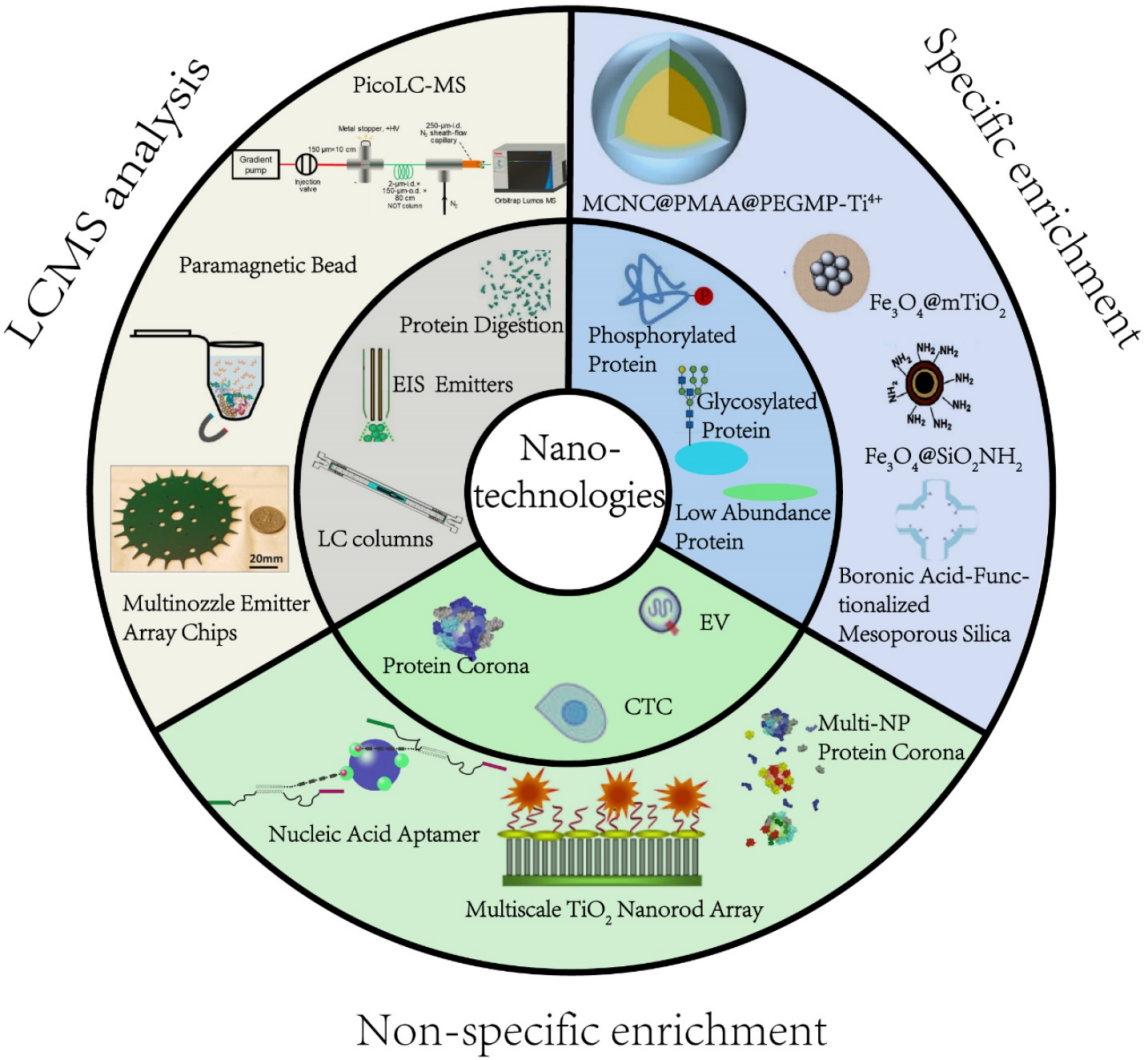
An overview of application of nanomaterials in proteomics-driven precision medicine (PDPM).

Efficient pretreatment methods are desired prior to MS analysis to reduce sample complexity and obtain deep proteome coverage. Nanostructured devices and nanoparticles (NPs) have unique characteristics, such as extremely small size, good biocompatibility, abundant active affinity sites, and large specific surface area, making them especially suitable for proteomic applications. For instance, magnetic nanocomposites have been used to purify proteins, significantly reducing the processing time. Mesoporous NPs, due to the adjustable pore size and large specific surface area, have been explored in size-based separation and membrane proteomics 12, 13. From the technical point of view of material science, several recent review articles have summarized the manufacturing of NPs and their applications in biomedical science 14. In this review, we intend to fill the gap from a proteomics perspective by focusing on the most pressing challenges currently facing MS-based proteomics **(Figure 1).** We illustrate how recent nanotechnological innovations, mainly the studies that were published within five years, have aided in addressing these challenges. We specifically detail significant advances in precision medicine development and provide our vision of the future for nanotechnology-enabled proteomics.

## 2. Nanotechnology-enabled targeted and non-targeted proteome analysis

Proteins can be analyzed on MS with full-length and intact forms, called top-down proteomics, or peptides after proteolytic digestion, also known as bottom-up or shotgun proteomics 15. Either way, proteins of interest must be isolated from the complex background before introducing into MS. For instance, protein acetylation is known to play a key role in regulating chromatin accessibility and gene transcription (*e.g.*, histones) as well as protein localization and enzymatic activity (*e.g.*, non-histone proteins) 16. To analyze protein acetylation using proteomic approaches, antibody-based enrichment is usually performed to separate acetylated proteins or peptides from non-acetylated matrix 17. Hence, signal suppression can be minimized or avoided during MS acquisition. Such targeted enrichment processes can significantly enhance the chance of identifying important but low-abundance modified proteins. Some NPs (e.g., iron and titanium oxide) have unique physiochemical properties and can specifically bind proteins with certain PTMs, such as phosphorylation and glycosylation, facilitating the identification of these events. On the other hand, other NPs can non-selectively bind proteins when they encounter biospecimens 18. Such non-targeted enrichment offers an interesting alternative to global proteome profiling. In the following sections, we summarize recent developments in these areas.

### 2.1 Specific enrichment of protein PTMs

Protein modifications play essential roles in a wide range of physiological and pathological processes 19. Information on protein abundance together with PTM alterations has been shown to uncover disease markers 20. From a purely technical perspective, the major challenge to study post-translationally modified proteins by MS is their low abundance 21. Here, we focus on the two most widely studied PTMs, phosphorylation and glycosylation.

**Figure 2:**
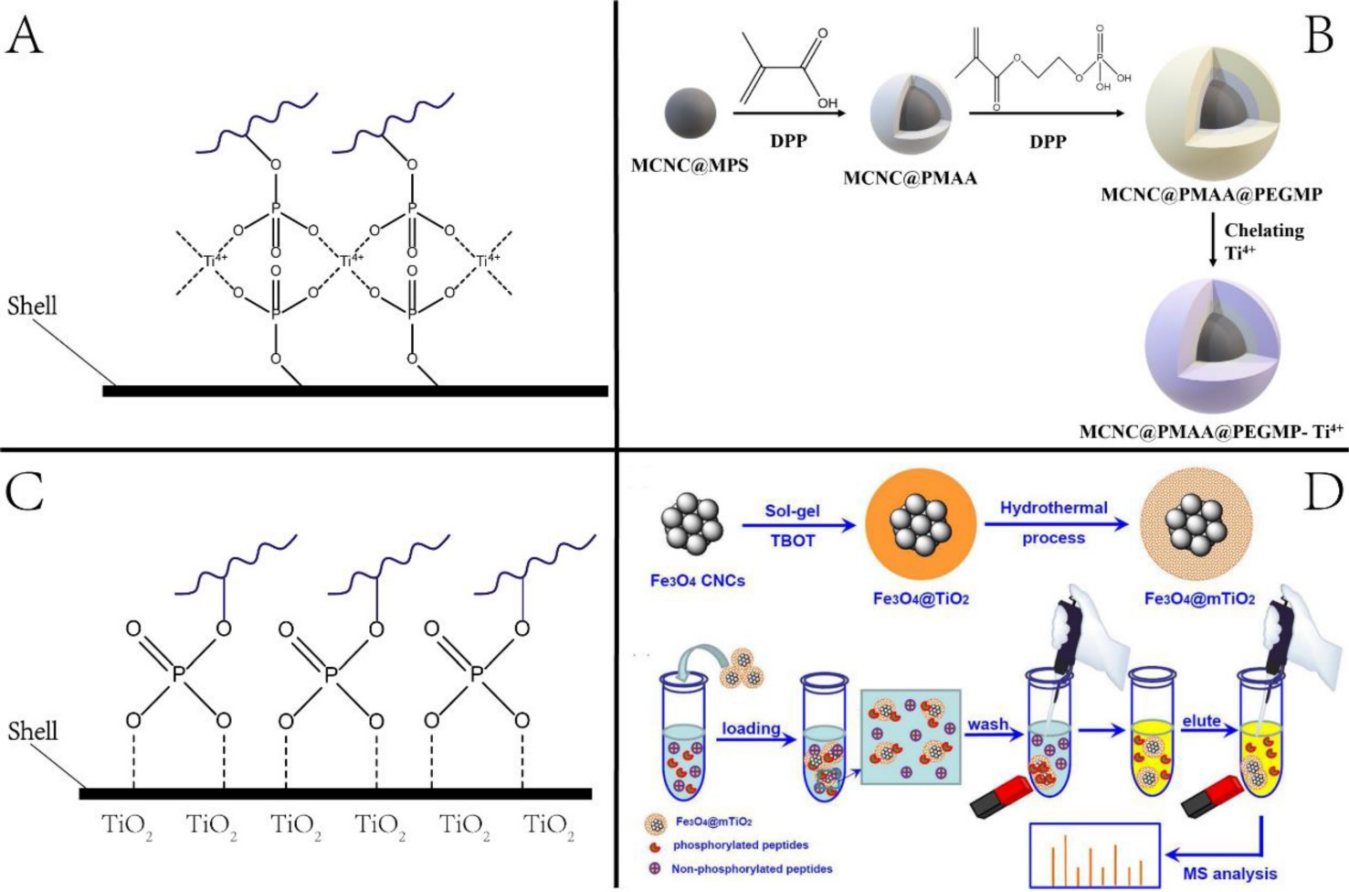
Nanotechnologies for enriching phosphopeptides. (A, B) Principle and example of phosphopeptides enrichment by Immobilized metal ion affinity chromatography (IMAC) method. (C, D) Principle and example of phosphopeptides enrichment by metal oxide affinity chromatography (MOAC) method. Adapted with permission from Ref. 27. Copyright 2012 American Chemical Society.]

**2.1.1 Phosphorylation** is recognized as one of the most common PTMs. It regulates protein function and cell signaling and plays a central role in disease-causing aberrations of kinase signaling networks 22. However, proteomic analysis of phosphorylation remains challenging due to its low stoichiometry. Magnetic NPs, such as immobilized metal ion affinity chromatography (IMAC) and metal oxide affinity chromatography (MOAC), have been widely used for phosphoproteomic studies 23. For the IMAC approach, metal ions, such as Al^3+^, Ga^3+^, Fe^3+^, Ti^4+^, Zr^4+^, are functionalized onto NPs. Since the metal ions are typical Lewis acids under acidic conditions, they can interact with phosphate groups through bridged two-dentate chelation and then capture the phosphopeptides. The principle of enrichment and a representative example are illustrated in **Figure 2**. The enrichment condition is usually adjusted to pH 2-3 to neutralize the acidic residues for avoiding non-specific adsorption of non-phosphopeptides with multiple acidic residues onto the IMAC or MOAC.

A large class of IMAC materials has been developed by varying the nature of immobilized metal and ligands to afford abundant metal sites 24. We previously synthesized Ti^4+^-immobilized magnetic composite microspheres using a high-magnetic-response magnetic colloid nanocrystal cluster (MCNC) core, a poly(methyl acrylic acid) (PMAA) interim layer, and a Ti^4+^-immobilized poly(ethylene glycol methacrylate phosphate) (PEGMP) shell (Figure 2) 25. Due to the pure phosphate-Ti^4+^ interface and high Ti^4+^ loading amount, the resulting MCNC@PMAA@PEGMP-Ti4+ composite microspheres demonstrated rapid (< 5 min) and efficient (phosphopeptide/nonphosphopeptide = 1:500) separation of phosphopeptides 25. As for the MOAC material, the main strategy for its synthesis was *in situ* growth of metal oxides on magnetic nanoparticles/microspheres 26. Porous structured MOAC was designed to improve the enrichment efficiency, as it has a large specific surface area and can offer abundant reaction sites for anchoring phosphopeptides 27. Except for TiO_2_, other nanomaterials with titanium have been reported that can be used to enrich phosphopeptides, but their application is limited, and is not discussed herein.

Application of the enrichment strategy to biomedical research, especially for biomarker discovery, is one of the most promising areas of the proteomics field. Jiang and coworkers recently applied titanium dioxide (TiO_2_) beads to enrich phosphopeptides from 110 paired tumor and non-tumor tissues of early-stage hepatocellular carcinoma (HCC). Nearly 30,000 phosphorylation sites were identified in total, and hyper-phosphorylation of signaling pathways in HCC was revealed 28. Chen *et al.* used Ti^4+^-functionalized dendrimer to enrich phosphopeptides from human plasma-derived extracellular vesicles. Among the over 10,000 unique phosphopeptides, they reported 144 phosphoproteins significantly high in breast cancer patients 29. These findings suggested that nanomaterial-based enrichment strategies can identify additional disease biomarkers compared to conventional shotgun proteomics.

**2.1.2 Glycosylation** is another important and most structurally complicated protein modification 30. Depending on the linkage sites, there are two common types of protein glycosylation, N-glycosylation, where glycans are attached to asparagines *via* N-acetylglucosamine residues, and O-glycosylation, where the glycans are attached to serine or threonine through acyl linkages. The cancer glycoproteome varies at different levels, including alterations in the glycosylation sites and/or glycan chain composition, offering tremendous potential for targeted therapeutics against glycosylation 31. There is growing evidence that cancer cells have a unique glycosylation profile, and alterations in glycosylation are regarded as a hallmark of cancer 32. Moreover, studies have shown that protein glycosylation can significantly improve the clinical value of classical biomarkers 33.

**Figure 3:**
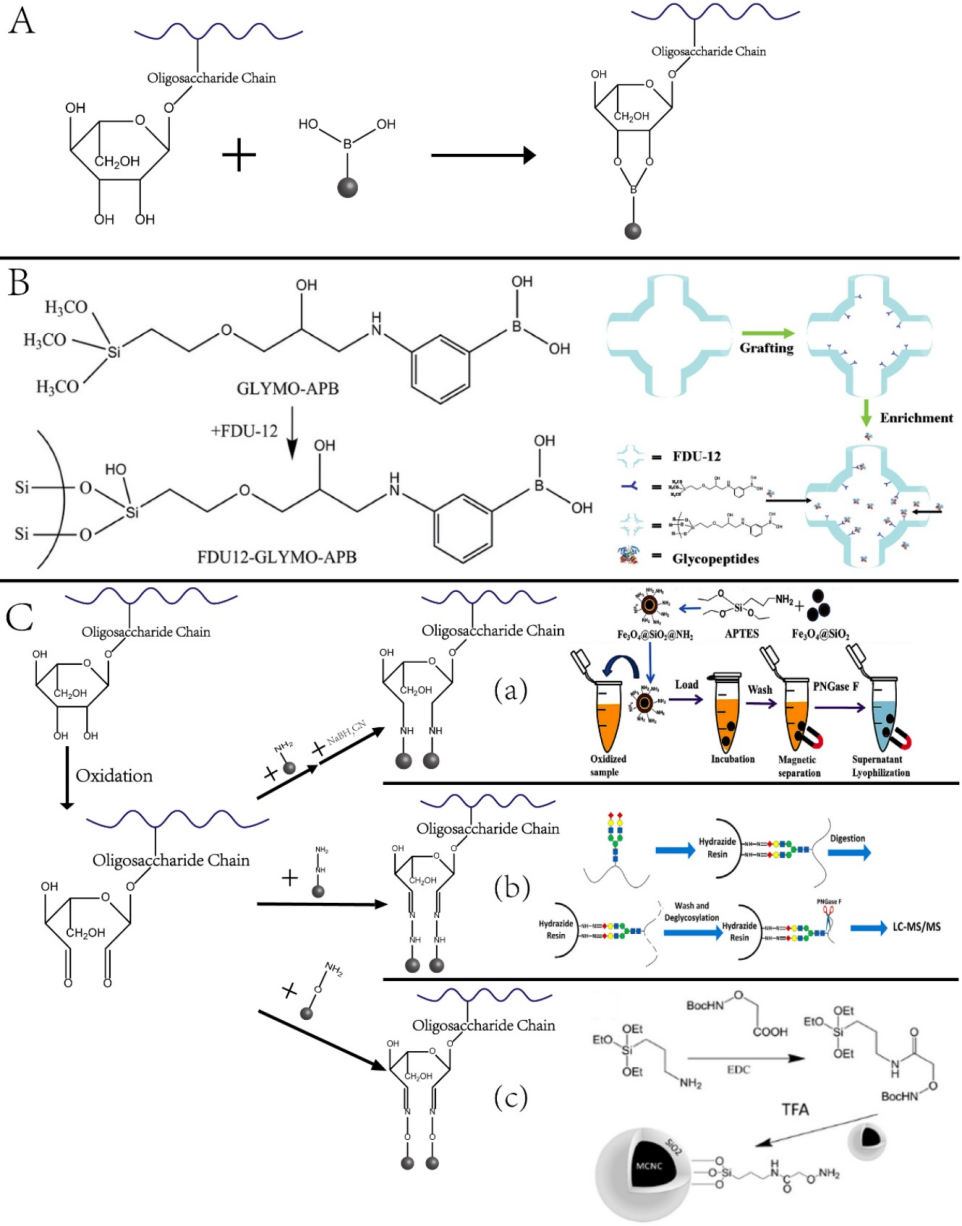
Nanotechnologies for enriching glycoproteins. (A, B) Principle and example of glycoproteins enrichment by boric acid functionalized materials. Adapted with permission from Ref. 34. Copyright 2009 American Chemical Society.] (C) Aldhyde-based covalent bond formation assisted enrichment methods. a) Amine-functionalized nanoparticles. [Adapted with permission from Ref. 35. Copyright 2013 American Chemical Society]. b) Hydrazide Chemistry. [Adapted with permission from Ref. 36 Copyright 2014 American Chemical Society] c) Oxime Click Chemistry. [Adapted with permission from Ref.^37^. Copyright 2014 American Chemical Society]

Glycosylation enrichment using nanomaterials is based on affinity-based and covalent binding-based enrichment. In affinity-based methods, lectin is usually immobilized onto the surface of NPs, and glycopeptides are enriched by hydrophilic interaction between the particles and sugar chains. In the method based on covalent interaction, glycopeptides are mainly enriched by the interaction between the boronic acid and the cis-diol-containing glycoproteins/glycopeptides 34 or the interaction between aldehydes produced by the oxidation of sugar chains and hydrazine, hydroxylamine, and other functional groups on the surface of solid materials 35-37. The principle and representative examples are displayed in **Figure 3.** As summarized in a recent review article, a variety of functional materials have been investigated, including magnetic and mesoporous materials, metal frame compounds, graphene, and dendrimers 38.

Our group designed and synthesized new aminooxy-functionalized magnetic nanoparticles using oxime click reaction-based enrichment 37. The oxidized glycan chains on the glycopeptides could conjugate with aminooxy groups through oxime click reaction, retaining the glycopeptides on the magnetic NPs. The oxime click chemistry-based method renders excellent enrichment performance within one hour, and the magnetic core brings the advantage of the fast separation of glycopeptide with good reproducibility. Notably, because cis-diol-containing glycoproteins/glycopeptides can form stable and reversible covalent bonds with boronate by adjusting pH, functionalized nano-systems using boronic acid have been established to enrich glycoproteins. A typical example is the fabrication of dendrimeric boronic acid-functionalized magnetic nanoparticles for enriching glycoproteins from a complex sample 39. Due to the dendrimer-assisted multivalent synergistic binding, the boronate avidity material exhibited more than three orders of magnitude higher dissociation constants than the affinities of single boronic acid binding towards glycoproteins, leading to significantly efficient enrichment of glycoproteins 36.

In this context, several recent glycoproteomic studies have reported novel glycoproteins that could serve as noninvasive biomarkers. From a cohort of 74 aggressive and 68 non-aggressive prostate cancer patients, a glycoproteomic study proposed a three-glycoprotein panel (ACPP, CLU, and PSA) that could distinguish between aggressive and non-aggressive prostate cancers with an AUC of 0.86 40. Pan et al. investigated the role of protein glycosylation in high-grade serous ovarian carcinoma (HGSC) by extracting glycoproteins from 119 TCGA HGSC tissues using two independent approaches, solid-phase extraction of glycoside-containing peptides (SPEG) and intact glycopeptides for investigating glycoside-specific glycans (IGPs). The study discovered that glycosylation with specific glycol-form features was associated with disease progression and severity, indicating that a deep understanding of the proteome glycosylation might provide important clues for precision medicine 41.

**2.1.3 Other PTMs.** Cysteine is prone to post-translational modifications because of its high reactivity, including affinity and redox sensitivity. Common PTMs on cysteine include redox-dependent nitrosylation (SNO), lipid-derived electrophilic 4-hydroxynonenal (HNEs), and isoprene lipid modifications. For example, a novel approach based on the fluorous solid-phase extraction of SNO-peptides using nanographite fluoride was developed to analyze the SNO-proteome. In this strategy, a fluorous tag, which could increase the ionization efficiency of SNO-peptides and facilitate the following enrichment, was introduced into the SNO modification sites. Subsequently, the fluorescence-tagged SNO-peptides were captured by nanographite fluoride specifically through fluorous-fluorous interactions. Taking advantage of the highly fluorinated level and the high surface area of nanographite fluoride, the enrichment approach was shown to have remarkable selectivity, good sensitivity, high post-enrichment recovery, and large enrichment capacity 42, 43.

### 2.2 Specific enrichment of low abundance proteins

Blood is one of the most common body fluids used for laboratory-based analyses. It contains a wide range of molecules, such as electrolytes, small molecules, drugs, and proteins, routinely tested in clinical settings. Although extremely challenging, MS-based proteomics is an unbiased way to identify novel blood biomarkers. The dynamic range of the blood proteome is more than ten orders of magnitude, and the top 10 proteins with the highest abundance account for nearly 90% of the total protein content 8. Therefore, without depletion, fractionation, and/or enrichment, only a few hundred proteins could be identified by LC-MS/MS 8, 44; hence, nanomaterials have found wide applications in blood proteomics. Stable Isotope Standards and Capture by Anti-Peptide Antibodies (SISCAPA) is a classical method based on antibody-immobilized magnetic nanomaterials to capture specific protein biomarkers from blood 45. Recent developments of the SISCAPA include automation 46, cost-effectiveness 47, and quantitation 48, 49. The latter included the recently developed targeted proteomics approach, multiple reaction monitoring (MRM), and demonstrated a lower incidence of false-negative findings in the early detection of hepatocellular carcinoma 49.

Another important application of specific enrichment of blood proteins using NPs is the recently designed surface-functionalized superparamagnetic iron-oxide (magnetite, Fe_3_O_4_), which specifically enriched a well-established cardiac biomarker troponin I (cTnI). The study analyzed cTnl using the top-down approach and demonstrated an association between molecular fingerprints of diverse cTnI forms and pathophysiology 50.

### 2.3 Non-specific enrichment of proteins and cells

While specific enrichment of proteins of interest has shown great selectivity and broad application in biomedical research, emerging non-specific enrichment approaches with NPs offer interesting alternatives to conventional global and targeted proteomic analysis.

**Figure 4:**
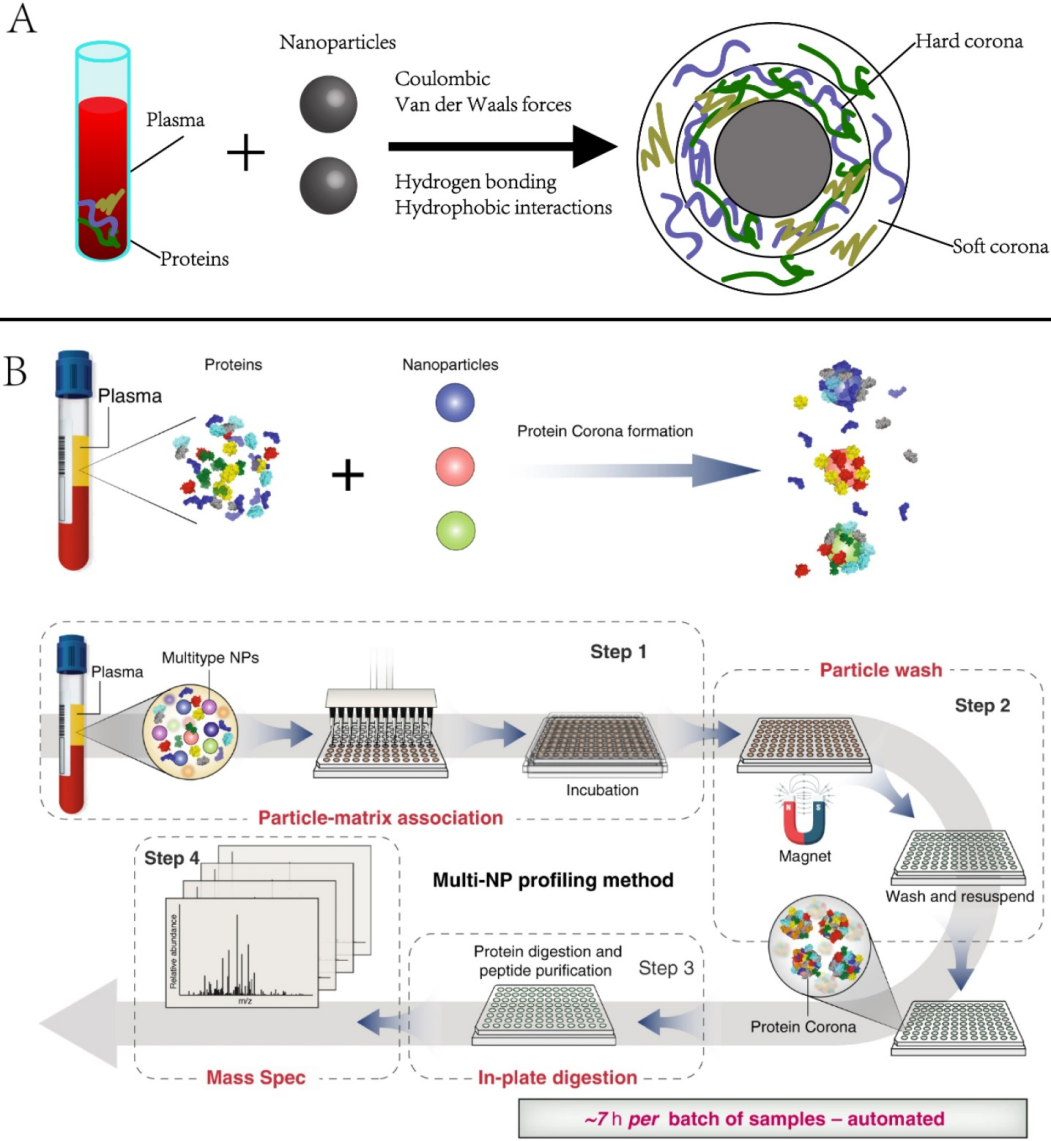
Principle and example of using nanoparticle protein corona in protein biomarker discovery. Adapted with permission from Ref. 53 Copyright 2020 Spring Nature.

**2.3.1 Protein corona.** Protein corona is the group of proteins that are spontaneously and non-specifically adsorbed to NP surfaces when they encounter biospecimens or are introduced to the physiological environment 51, 52. It alters the physicochemical properties of NPs and eventually affects their circulation, bio-distribution, and toxicity in living systems. Numerous studies have investigated the physicochemical properties (*e.g*., size, shape, and coating) of NPs in the context of *in vivo* imaging, drug delivery, or targeted therapies 53-56. However, the protein corona has not been often used for biomarker discovery. Recently, Blume *et al.* reported comprehensive profiling of the protein corona pulldown by NPs, representing a subset of the plasma proteome. 53. As the protein corona varies with NP properties, combining multiple different types of engineered NPs is expected to capture distinct protein corona patterns, enabling deep profiling of plasma proteome **(Figure 4).** The investigators screened 43 types of magnetic nanoparticles and demonstrated that using a panel of 10 could achieve in-depth plasma proteome profiling across more than seven orders of magnitude, including the identification of 53 FDA-approved protein biomarkers in a single pooled plasma. They further applied the panel to profile the plasma proteome of early-stage non-small-cell lung cancer (NSCLC) patients and age- and gender-matched healthy controls. A multi-protein classifier was identified to distinguish NSCLC patients from healthy controls with a high average area under the curve (AUC) of 0.91

Although proteins are the major components of the NP corona, recent studies revealed that non-protein entities, such as saccharides and lipids, could also be detected in the corona 57. Therefore, multiomics investigation of the NP corona would enable a better understanding of its composition and biological identity, eventually enabling precise NP designs and the development of safe nanomedicines.

**2.3.2 Circulating tumor cells (CTCs).** CTCs are shed into the circulation from primary or metastatic tumors. Documented evidence has suggested that detection of CTCs in peripheral blood offers a unique opportunity to identify prognostic markers for various cancers and better understand cancer metastasis and tumor heterogeneity 58, 59. The major challenge for CTC detection is their extreme paucity (1-10 cells per 10 ml peripheral blood) 59. Conventional protein-based technologies utilize antibodies to detect specific CTC markers. Immuno-magnetic beads (IMB) represent another promising alternative. Nano-sized beads have a higher specific surface area and thus higher cell separation efficiency than micron beads but may cause accumulation after high-speed centrifugation. Non-magnetic beads, such as TiO_2_ nanorods (160-300 nm diameter) on the F-doped SnO_2_ (FTO) substrate have also been examined 60. It is noteworthy that surface proteins on CTCs may change during the circulation, giving false-negative results. Furthermore, CTCs and normal cells have been shown to have different adhesion properties on rough surfaces. Therefore, a recent study attempted to fabricate glass surfaces with different roughness using ion etching, enabling enrichment of CTCs because of their higher adhesion to the surface than regular cells 61.

Recent studies also investigated the size-based separation of CTCs. The surface bombardment of polymer materials (*e.g.*, polycarbonate film and polyp-xylene film) by micromachining can form nanometer to micron size holes. Since the size of CTCs is usually around 20 μm, they can be effectively separated by microfiltration, for instance, using 6-10 μm pore size membranes 62. However, uneven distribution of the pore size limits the capture efficiency of CTCs to only 50-60%. Yeh *et al.* managed to overcome the problem by fabricating a tandem flexible micro spring array (tFMSA) with micro mold. The resulting micro spring structure could separate CTCs from normal cells based on the size difference and deformability, thus increasing the CTC capture efficiency to 90%. Moreover, the study showed 86% of captured species were CTCs by measuring the marker of mesenchymal cells, vimentin, highlighting the advantage of microfilter separation 63. Other types of materials, such as graphene oxide (GO) and spiral microfluidics, have also been demonstrated to isolate CTCs from the blood 61, 62. In summary, NP-based enrichment of CTCs avoids potential losses of CTCs due to non-specific binding to antibody-coated beads and the resulting cytotoxicity. Thus, compared to traditional immunoaffinity-based and size-based filtration methods, NP-based CTC enrichment appears to have a broader application in biomedical research.

**2.3.3 Extracellular vesicles.** Nearly all cell types secrete lipid bilayer membrane-encapsulated vesicles ranging in diameter from 30 to 300 nm, referred to as extracellular vesicles (EVs) 64. Besides specific membrane lipids, EVs carry DNA and various RNAs, including miRNAs and proteins. EVs have diverse functions in immune tolerance 65, possess the ability to either promote or suppress cancer and promote the transfer of antimicrobial resistance proteins and genes 66, 67. Similar to the CTCs, EVs can be isolated by immunoaffinity-based methods using aptamers, short single-stranded nucleic acid sequences that function like antibodies but are economical, stable, and easy-to-modify, as demonstrated by recent EV separation studies 68, 69.

EVs can also be isolated by chelation. For instance, Sun *et al.* developed bifunctional magnetic beads (BiMBs) with a hydrophilic phosphate head for Ti^4+^ binding. The synergistic effect of Ti^4+^ with phosphate groups and 1,2-distearoyl-sn-glycero-3-phosphorylethanolamine enabled BiMBs to capture EVs from urine samples efficiently. Compared to conventional methods, BiMBs showed a much higher enrichment efficiency and lower sample consumption 70. Also, EVs could be separated free of antibodies based on their size by centrifugation. However, ultracentrifugation is time-consuming and requires expensive equipment. Studies that incorporated nano-membranes with different pore sizes during centrifugation 71 and utilized tangential flow filtration (TFF) technology solved the problem to some extent 72. Nevertheless, an inherent drawback of the size-based separation methods is that the isolated EVs may contain impurities of similar size. Therefore, the size-based separation techniques are often employed with other methods to achieve better separation results.

## 3. Nanotechnology and nanofabrication facilitate LC-MS/MS analysis

### 3.1 NP-assisted protein digestion

Digestion of biological samples is an important processing step upstream of the shotgun proteomic analysis for biomarker discovery and subsequent clinical development 73. Nanomaterials and/or microparticles are useful in facilitating proteomic sample digestion, especially for low-abundance proteins and those from protein-protein interaction complexes 74, 75. Several studies have investigated the on-bead digestion methodology in which the enriched proteins from cell lysate are digested directly on the magnetic 76, 77, chromatographic 78, or agarose beads 79. Hughes *et al.* developed a single-pot solid phase-enhanced sample-preparation technology, termed SP3, which utilizes interactions (*e.g.*, hydrophilic, electrostatic repulsion) between proteins and the carboxyl or amine group of functionalized paramagnetic beads 74. Proteins can be non-selectively captured onto the beads, and after simple rinse and wash, are digested directly with minimal sample loss. Such one-pot, solid-phase-based methods tend to have fewer liquid handling steps and, therefore, are faster and more efficient than conventional gel-based and in-solution processing. This approach has identified novel interacting proteins and mechanisms to further our understanding of pathogenesis, such as in protein-misfolding disease and tumor regression 79, 80. Recently, this technique was implemented on a liquid handling robot for automated processing of samples in a 96-well format 81 and showed high sensitivity and good reproducibility in analyzing serum/plasma 82, FFPE tissues 83, and cell lines 84.

Immobilized enzyme reactor (IMER) has also been employed extensively to pre-treat samples for proteomic profiling 85. Generally, it uses a capillary column as a packing bed for NPs and immobilizes proteolytic enzymes via chemical coupling. Recently, covalent organic frameworks have been reported as a platform for enzyme immobilization 86. As the immobilization process reduces diffusion distance and increases the local enzyme concentration, the IMER significantly shortens the digestion time from hours to minutes and speeds up the entire proteomic analysis pipeline 85, 87. Fan et al. recently synthesized a thermo-responsive magnetic fluid (TMF) to expand the IMER application beyond classical tryptic digestion by immobilizing trypsin and the PNGase enzyme, enabling highly rapid and efficient deglycosylation 88.

Another interesting application of NPs for proteomics sample treatment is the proteome reactor. NPs with different chromatographic properties, for instance, reversed-phase, ion exchange, are immobilized or packed into pipette tips, which could be used directly for cell lysis, protein digestion, and peptide fractionation 89-91. Applications of such stop-and-go-extraction tips (StageTips) include analyses of global proteome 89, glycoproteome 91, interaction proteome 92, and phospho-proteome 93.

### 3.2 Electrospray ionization emitters

Liquid chromatography-MS (LC-MS) is central to any proteomic investigation 94, and electrospray (ESI) is the interface between the two systems, introducing biomolecules into the MS 95. A variety of ESI emitters of different shapes (*e.g.*, tapered vs. non-tapered) and sizes (*e.g.*, wide vs. narrow openings) have been developed, aimed at maximizing the transferring efficiency 96, 97. However, emitters are prone to clogging, especially for nanoESI. Another method, nanoflow LC that typically flows at 50-500 ml/min, has routinely been used for protein identification due to its high ionization efficiency and hence better sensitivity than ESI-MS 73. However, it lacks robustness and throughput for large-scale and complex proteome analysis 98. In contrast, microflow LC-MS with a flow rate between 1-50 μl/min usually shows robustness but at the cost of ionization efficiency and sensitivity 99. The microfabricated monolithic multi-nozzle emitter is a design that splits the microflow into multiple nanoflows, and, therefore, offers advantages of both microLC and nanospray in one LC-MS run, thereby boosting ionization stability and efficiency 100.

### 3.3 NanoLC columns

The separation efficiency of LC is one of the determining factors for the overall proteomic analysis. Although microcapillary columns have been mostly used, studies have shown that smaller inner diameters (i.d.) (from 75um to 30 um) could increase protein identification by 95% 101, 102. Microfabricated pillar array columns 102 and porous-layer open tubular columns 103 have been demonstrated to improve separation efficiency and increase proteome coverage for low-input samples. Recently, Xiang *et al.* reported that a 2-um i.d. narrow open tubular column running at picoflow (< 800 pl/min) could identify nearly 1,000 proteins from less than 100 pg tryptic digests 102. Also, Wang *et al.* reported a segmented microfluidic method to pack ultralong up to 5 m capillary columns 104. In contrast to the packed-bed capillary columns, a micro-pillar array column (μPAC) has recently been demonstrated to be more efficient than particle-packed columns, achieving up to one million theoretical plates at specific conditions 105. The μPAC leads to much lower back pressure, contributes to higher peak capacity, and identifies a greater number of peptides and proteins from complex samples.

## 4. Conclusions and perspectives

The focus of this article was to summarize recent advances in nanotechnology-enabled proteome analysis and its applications to precision medicine development. Important aspects, such as the design and preparation of nanoparticles, the versatility of functionalization methods, and enrichment principles and efficiency, were discussed. Some of the proteomic applications by coupling various nano-techniques to mass spectrometry are summarized in Table 1. Due to space limitations, regrettably, other similar studies could not be included in the present review.

As discussed above, nanomaterials have distinct advantages over conventional assays, such as good biocompatibility, abundant active affinity sites, large specific surface area, and unique surface properties, providing a combination of important features for proteomic analysis. Currently, enrichment of low abundance proteins, protein modifications, and miniaturized devices for sample preparation are major areas that could benefit from the application of NPs. Although most enrichment processes are carried out *in vitro*, in the future, the development of *in vivo* enrichment technologies, linking the spatial location and functional information of proteins, and providing more accurate information for precision medicine development can be envisioned. With the emerging roles of protein modifications in human diseases 106-108, nanotechnologies with new types of materials, functionalization methods, and reagent generation will continue to play key roles in identifying and enriching modified proteins. Moreover, single-cell proteomics is emerging as a promising tool to uncover the heterogeneity between cells and identify new cell subtypes 109. However, single-cell proteomics faces extreme challenges for in-depth global analysis and investigation of their modifications. The unique surface properties and large specific surface area of nanomaterials to capture the proteome in single-cell samples with high efficiency offer advantages for developing sensitive, simple, and rapid detection methods necessary for proteomics-driven precision medicine.

## Figures and Tables

**Table 1:** Summary of the nanotechnologies in PDPM discussed in this review.

Nanotechnology	Target	Type of technology and material		Application	Main findings	Ref.
Specific enrichment	Phosphorylated protein	Immobilized metal ion affinity chromatography (IMAC)	Ti^4+^ -immobilized magnetic composite microspheres	Milk and Human Serum	Efficient separation phosphopeptide/nonphosphopeptide = 1:500)	25
		Metal oxide affinity chromatography (MOAC)	Titanium dioxide (TiO_2_) beads	Liver proteins	Nearly 30,000 phosphorylation sites and several hyper-phosphorylation of signaling pathways in HCC	28
			Ti^4+^-functionalized dendrimer	Plasma Microvesicles and Exosomes	144 phosphoproteins among the over 10,000 unique phosphopeptides are significantly higher in patients diagnosed with breast cancer compared with healthy controls	29
	Glycosylated protein	Magnetic nanomaterials	Aminooxy-functionalized magnetic nanoparticle	Asialofetuin from fetal calf serum and myoglobin from horse heart	Highly efficient separation of N-glycoproteins with excellent sensitivity and be able to effectively analyze a small sample	37
		Boronic acid functionalized nano systems	Dendrimeric boronic acid-functionalized magnetic nanoparticles	Human saliva	Exhibited a strong avidity towards glycoproteins, which was 3-4 orders of magnitude higher than the conventional boronate affinity of a single binding.	39
	SNO-proteome	Fluorous solid-phase extraction	nanographite fluoride	Human Umbilical Vein Endothelial Cell (HUVEC)	Better selectivity, lower limit of detection, and higher post enrichment recovery as well as large enrichment capacity.	42, 43
	Low abundance protein	Antibody-immobilized magnetic nanomaterial	Stable Isotope Standards and Capture by Anti-Peptide Antibodies (SISCAPA)	Human plasma, human mammary epithelial cell line	Can enrich specific peptides from a mixture, the antibody-coupled beads can be reused consistently for up to 10 times.	45-49
		Magnetic nanomaterials	Surface-functionalized superparamagnetic iron-oxide (magnetite, Fe_3_O_4_)	Human plasma	Specifically enrich cardiac troponin I (cTnI), a well-established cardiac biomarker	50
Non-specific enrichment	Protein corona	Magnetic nanomaterials	43 types of magnetic nanoparticles	Human plasma	10 of them can achieve efficient plasma proteome profiling across more than seven orders of magnitude, including the identification of 53 FDA-approved protein biomarkers in a single pooled plasma.	53
	Circulating tumor cells (CTCs)	Non-magnetic nanomaterials	TiO_2_ nanorods (160-300 nm diameter) on F-doped SnO_2_ (FTO) substrate	Artificial blood samples	Can effectively enhance the capture performance of target cancer cells even in a low cell density situation.	60
		Nanomaterials with different size	Tandem flexible micro spring array (tFMSA)	Human blood	Increasing the CTCs capture efficiency to 90%	63
	Extracellular Vesicles	Magnetic nanomaterials	Bifunctional magnetic beads (BiMBs)	Urine	Higher enrichment efficiency and lower sample consumption	70
		Tangential flow filtration (TFF)	nano-membranes	Undiluted Serum	Operating pressures are orders-of-magnitude lower than membranes with conventional thicknesses (1-10 µm). Captured particles are associated with a surface, rather than trapped in a bulk-matrix. Higher efficiency of capture and release of particles.	72
LCMS analysis	Protein digestion	Magnetic, chromatographic or agarose beads	Functionalized paramagnetic beads	*Drosophila* embryo samples	Presented a novel protocol using paramagnetic beads, Single-Pot Solid-Phase-enhanced Sample Preparation (SP3).	74
	Electrospray ionization emitters	Nanoflow LC	Microfabricated monolithic multinozzle emitter	Low-volume whole blood samples	Achieved a detection limit of less than 5 red blood cells	100
	LC columns	Microfabricated pillar array columns	A 2-um i.d. narrow open tubular column running at picoflow (< 800 pl/min)	Tryptic peptides	Provide nearly 1,000 protein identifications from only less than 100 pg tryptic digests	102
			Micro-pillar array column (μPAC)	Tryptic digest of a mixture of seven proteins with diverse mass and isoelectric point	High flow rates (600 nl/min) and higher protein identification rates.	105
